# Supplementary material for: Evaluation of health-related quality of life and the related factors in a group of Chinese patients with interstitial lung diseases
Source: PLoS One. 2020 Jul 29;15(7):e0236346. doi: 10.1371/journal.pone.0236346 (PMC7417083; doi:10.1371/journal.pone.0236346)
Supplement: S1 Table — (DOCX) [file pone.0236346.s001.docx]

**S1 Table. IIP and CTD-ILD subgroups.**

| IIP subgroups | |
| --- | --- |
| Idiopathic NSIP | 101 |
| Unclassifiable IIP | 18 |
| Respiratory-bronchiolitis-ILD | 11 |
| IPF | 7 |
| Others* | 2 |
| CTD subgroups | |
| SS | 12 |
| Undifferentiated CTD | 6 |
| PM/DM | 4 |
| SSc | 3 |
| RA | 2 |
| Others** | 3 |

Data are presented as numbers of cases. IIP, idiopathic interstitial pneumonia; CTD, connective tissue disease; ILD, interstitial lung disease; NSIP, non-specific interstitial pneumonia; IPF, idiopathic pulmonary fibrosis; SS, Sjögren’s syndrome; PM/DM, polymyositis/dermatomyositis; SSc, scleroderma; RA, rheumatoid arthritis.

* for IIP, the others subgroup includes cryptogenic organizing pneumonia and acute interstitial pneumonia; ** for CTD, the others subgroup includes cytoplasmic antibody-associated vasculitis, eosinophilic granulomatosis with polyangiitis, and IgG4-related disease.
