# Supplementary material for: Evaluation of health-related quality of life and the related factors in a group of Chinese patients with interstitial lung diseases
Source: PLoS One. 2020 Jul 29;15(7):e0236346. doi: 10.1371/journal.pone.0236346 (PMC7417083; doi:10.1371/journal.pone.0236346)
Supplement: S2 Table — (DOCX) [file pone.0236346.s002.docx]

**S2 Table. Associations between changes in HRQoL and clinical characteristics.**

| Variable | Value (6-month follow-up) | | |  | Value (12-month follow-up) | | |
| --- | --- | --- | --- | --- | --- | --- | --- |
|  | n | Change in SGRQ total | P value |  | n | Change in SGRQ total | P value |
| Change in the FVC% predicted |  |  | 0.000 |  |  |  | 0.958 |
| Improvement >10% predicted | 9 | -24.8±20.7 | 0.000 |  | 10 | 0.6±22.9 | 0.612 |
| Improvement >5 to ≤10% predicted | 9 | -21.2±15.2 | 0.003 |  | 11 | -2.2±17.7 | 0.842 |
| Improvement >2 to ≤5% predicted | 13 | -9.9±10.4 | 0.322 |  | 18 | -6.9±21.6 | 0.699 |
| Stable | 24 | -5.5±10.4 | - |  | 14 | -3.9±20.8 | - |
| Decline >2 to ≤5% predicted | 15 | -3.1±13.3 | 0.576 |  | 9 | -7.1±13.8 | 0.728 |
| Decline >5 to ≤10% predicted | 6 | 18.0±11.3 | 0.000 |  | 12 | 0.0±27.9 | 0.642 |
| Decline >10% predicted | 5 | 9.1±8.8 | 0.025 |  | 5 | -0.9±18.5 | 0.790 |
| Change in the DLco% predicted |  |  | 0.000 |  |  |  | 0.000 |
| Improvement >10% predicted | 19 | -18.5±17.8 | 0.004 |  | 20 | -17.9±22.7 | 0.188 |
| Improvement >5 to ≤10% predicted | 6 | -17.0±15.2 | 0.099 |  | 7 | -7.3±12.0 | 0.017 |
| Stable | 35 | -6.3±12.9 | - |  | 23 | -4.3±13.5 | - |
| Decline >5 to ≤10% predicted | 6 | 13.1±12.9 | 0.004 |  | 9 | 11.7±24.1 | 0.000 |
| Decline >10% predicted | 11 | 5.2±13.9 | 0.025 |  | 14 | 12.1±15.5 | 0.000 |
| Change in the mMRC |  |  | 0.000 |  |  |  | 0.000 |
| Improvement ≥1 | 32 | 8.0±13.3 | 0.000 |  | 44 | 11.7±19.6 | 0.000 |
| Stable | 83 | -4.6±12.5 | - |  | 64 | -5.7±13.2 | - |
| Decline ≤1 | 31 | -21.2±18.5 | 0.000 |  | 32 | -22.7±17.8 | 0.000 |
| Change in the total LCQ total score |  |  | 0.000 |  |  |  | 0.000 |
| Improvement ≥1.3 | 62 | -13.2±19.2 | 0.000 |  | 54 | -5.0±21.6 | 0.001 |
| Stable | 55 | -1.9±11.2 | - |  | 50 | -2.2±12.9 | - |
| Decline ≥1.3 | 30 | 4.5±13.6 | 0.071 |  | 36 | 9.5±19.6 | 0.004 |

Data are presented as a number or the mean±SD. FVC, forced vital capacity; DLco, diffusing capacity of the lung for carbon monoxide; mMRC, modified Medical Research Council dyspnea scale; LCQ, Leicester Cough Questionnaire
