# Supplementary material for: Evaluation of health-related quality of life and the related factors in a group of Chinese patients with interstitial lung diseases
Source: PLoS One. 2020 Jul 29;15(7):e0236346. doi: 10.1371/journal.pone.0236346 (PMC7417083; doi:10.1371/journal.pone.0236346)
Supplement: S3 Table — (DOCX) [file pone.0236346.s003.docx]

**S3 Table. Comparison of the main demographics, clinical characteristics and HRQoL according to the prognosis.**

| Characteristics | Non-Survivors | Survivors | P value |
| --- | --- | --- | --- |
| Number of deaths | 16 | 153 | —— |
| Age, years | 66.7±8.3 | 63.6±11.1 | 0.448 |
| Male sex | 10 (62.50%) | 81 (52.9%) | 0.229 |
| Duration since the first symptoms in months | 24.0 [12.0, 24.0] | 12.0 [2.0, 30.0] | 0.077 |
| ILD subtypes |  |  | 0.181 |
| IIP | 15 (93.80%) | 124 (81.0%) |  |
| CTD-ILD | 1 (6.20%) | 29 (19.0%) |  |
| Cause of death |  |  | —— |
| Pulmonary infection | 10 (62.50%) | —— |  |
| AE-ILD | 5 (31.25%) | —— |  |
| Lung cancer | 1 (6.25%) | —— |  |
| HRQoL |  |  |  |
| SF-36 PCS | 27.1 [17.6, 32.8] | 38.1 [29.8, 46.9] | 0.000 |
| SF-36 MCS | 52.5 [46.3, 63.0] | 49.3 [41.7, 56.4] | 0.159 |
| SGRQ Symptom | 52.6 [35.6, 79.6] | 34.0 [15.4, 55.0] | 0.029 |
| SGRQ Activity | 70.5 [53.7, 80.2] | 36.4 [18.3, 53.5] | 0.000 |
| SGRO Impact | 47.06 [29.9, 58.1] | 22.0 [13.7, 37.2] | 0.001 |
| SGRQ Total | 58.3 [36.7, 66.7] | 28.5 [19.0, 44.3] | 0.000 |

Data are presented as a number (%) or the mean±SD. ILD, interstitial lung disease; IIP, idiopathic interstitial pneumonia; CTD, connective tissue disease; CTD-ILD, CTD-associated ILD; AE-ILD, acute exacerbation of ILD; HRQoL, health-related quality of life; SF-36, the Medical Outcomes Study Short Form 36; PCS, physical component score; MCS, mental component score; SGRQ, St. George’s Respiratory Questionnaire
