# Supplementary material for: Evaluation of health-related quality of life and the related factors in a group of Chinese patients with interstitial lung diseases
Source: PLoS One. 2020 Jul 29;15(7):e0236346. doi: 10.1371/journal.pone.0236346 (PMC7417083; doi:10.1371/journal.pone.0236346)
Supplement: Attachment — Submitted filename: Response to Reviewers.docx [file pone.0236346.s004.docx]

Dear Prof. Paola Faverio,

Thank you for your kind letter. We revised the manuscript in accordance with your and the reviewers’ comments and advices, and carefully proof-read the manuscript to correct spelling, expression and statistical analytical approach errors. Here below is our description on revision according to your and the reviewers’ comments and advices.

Thank you for your submission to PLOS ONE. Both the reviewers highlighted the originality of the data and the importance of the topic. However some fundamental methodological pitfalls need to be addressed.

In particular, the heterogeneity of the population analysed. Given the high number of diagnoses, with different clinical characteristics and prognosis, that can be included in the family of interstitial lung diseases, I suggest to restrict the study to the main diagnoses. Furthermore, the statistical analysis need a complete revision by an expert statistician.

We appreciate all the comments and suggestions from the editors and reviewers, these will be great of help to improve our present work. We appreciate the suggestion and totally agreed with your opinion about the heterogeneity of the population. We restrict the population in our study to idiopathic interstitial pneumonias (IIP) and CTD-ILD, and then we reanalyzed the data. We also appreciate your reminding about the statistical analysis. We specifically consulted the expert statistician and improved the defects in our study.

Reviewer #1:

General comments

1. The researchers have collected an impressive amount of data, but the study lacks both structure and focus. The analytical approach is questionable and there are methodological limitations, in particular with respect to the study population. The manuscript contains large amounts of information, but adds little new knowledge. It is not easily accessible to the reader.

Response: Thank you for the comments. We agreed with the reviewer’s opinion on the defects in data processing and statistical analysis. The data of the study were from patients with ILDs in china, and there is a large space for study on the quality of life in these patients in china. In present study, We adjusted the study population to focus on patients with IIP and CTD-ILD, and we revised the writing of the manuscript to make it easier for the reader to access the important information (Line 236-243, Line 282-294, Line 444-452, Table 1, Table 2 ).

2. My main concern is the heterogeneous patient population. The material comprises a mix of diagnoses including idiopathic interstitial pneumonias (IIP), hypersensitivity pneumonitis (HP), CTD-ILD and sarcoidosis - all well-defined diseases with disease-specific clinical characteristics and disease-specific prognosis. To analyze such a variety of diseases under the same ILD-umbrella is not meaningful.

Response: Thank you for the comments. At first, we look forward to exploring a universal predictive model for ILDs, so we collected all patients clinically diagnosed with ILDs and followed them up for 12 months. Since ILD belongs to a group of diseases and the heterogeneity is very significant, it is obviously inappropriate. We think your views on the heterogeneous patient population are very reasonable and Based on your suggestion, we have eliminated HP, sarcoidosis and other types of ILD, and re-analyzed the data (Line 236-521, Table 1, Table 2, Figure 1, Figure 3 ).

3. The study lacks an appropriate purpose. The aim is to “investigate HRQL in patients with ILD and identify factors influencing HRQL among these patients”. To what end? What do the researchers want to find out? What is the hypothesis? ILD is a common denominator for various diseases with different courses, different responses to therapy and different prognosis. To assess HRQL in a mixed ILD-population is not useful. I would advise the research group to focus on disease-specific HRQL. Based on the number of patients in the various disease-groups, it may be an idea to confine the study to the two largest groups - IIP (69.5%) and CTD-ILD (15%) - and compare HRQL in those two diseases.

Response: Thanks for your comments and suggestions on our study. We agreed with the opinion on the aim of the study, and we focused on IIP and CTD-ILD according to your suggestion. We re-analyzed sociodemographic, disease-related characteristics in both ILD subtypes and compared HRQL in them.

4. The study lacks generalizability. The results of the study may be relevant to the Chinese medical community, but they cannot be generalized to other parts of the world. The study was carried out in 2017/18. At that time, anti-fibrotic treatment was commonly prescribed to patients with IPF in North-America, Europe, Australia and Japan. In the present study, only 5 patients received such drugs (table 1) while “other drugs” were used by 74% – presumably drugs that are not generally prescribed outside of China. Since drugs will affect symptoms, course of disease, side-effects and prognosis, they will also invariably affect measures of HRQL. This adds to the difficulties in interpreting and generalizing the results of the study.

Response: We appreciate your comment on the generalizability of the research. We are sorry that our way of expression may has interfered with you. In our study, “others” included “no intervention”, “antioxidants” and “traditional Chinese medicine”, which “no intervention” was used in 113 patients (56.2%). We refined the presentation of data to separate “no intervention” from “others” (table 1). Meanwhile, anti-fibrotic treatment is not commonly prescribed to patients with IPF due to the price problem, and anti-fibrotic treatment is only applicable to patients with IPF, not all patients with ILDs. Theses factors finally leads to the low application of anti-fibrotic treatment in present study. We agreed with your opinion that drugs may be the confounding factor affecting HRQL in ILDs. In present study, we focused on the impact of disease severity on quality of life. Although drugs may have an impact on quality of life, it is mainly a change in the severity of the diseases after treatment. We analyzed the effect of drugs factor on HRQL with ILD by linear regression analysis (table 3), but we are sorry that the statistical analysis cannot fully explain the real-world problems.

Specific comments

Introduction.

Lines 53-64, 65-67 and 67-68. Three statements that all need references.

Response: We appreciate for your advice, several publications have been added in corresponding statements of the article (**Line 67-68**, Curr Opin Pulm Med. 2013; 19: 474–479, Sarcoidosis Vasc Diffuse Lung Dis. 2016; 33: 341–348, Respirology. 2014; 19: 1019–1924; **Line 69-72**, Am J Respir Crit Care Med. 2007; 176: 636–643; **Line 74-75**, [Respir Res.](https://www.ncbi.nlm.nih.gov/pubmed/28709421) 2019; 20: 59–72, [Respirology](https://www.ncbi.nlm.nih.gov/pubmed/?term=Health-related+quality+of+life+in+idiopathic+pulmonary+fibrosis%3A+Data+from+the+Australian+IPF+Registry). 2017; 22: 950–956, Respir Res. 2020; 21: 36–47; **Line 79-80**, Respirology. 2014; 19: 1019–1924, [Respiration.](https://www.ncbi.nlm.nih.gov/pubmed/?term=Relationship+between+symptoms+and+quality+of+life+in+a+sarcoidosis+population) 2007; 74: 401–405; **Line 81-84**, Respir Med. 2017; 127: 1–6, Respirology. 2018, Chest. 2014; 145: 1333–1338, J Bras Pneumol. 2010; 36: 562–570).

Methods.

1. The prospective cohort was followed up though “face-to-face or telephone interviews”. How could “physiological indicators” (like PFTs) be registered by telephone? What was the procedure for follow-up with respect to the questionnaires? (lines 84-87)

Response: Thank you for your comments, it is indeed our unclear expression in previous manuscript. During the follow-up, some patients did not complete the questionnaires for personal reasons after pulmonary function test in our hospital. We registered the questionnaires by telephone within one week after the completion of the pulmonary function test. We corrected the inaccurate expression (Line 103-107).

2. ILDs have insidious onset. How was “date of ILD-diagnosis” defined? (line 96)

Response: Thank you for the remind. “Date of ILD-diagnosis” was defined as the time from symptom onset to the diagnosis of ILD in our hospital. We are sorry for the ambiguous expression and modified “Date of ILD-diagnosis” to “disease duration” (Line 116).

3. Equipment, guidelines and ref.values for PFTs should be specified. (lines 98-100)

Response: Thank you for your advice, we added relevant information in the manuscript (Line 123-128).

4. Were the two radiologists blinded to clinical information? (line 101)

Response: Yes, we are sorry for our negligence of this information and added it into the manuscript (Line 129-131).

5. The minimum clinically important differences in scores should be given for each of the questionnaires.

Response: Thank you for the remind, the minimum clinically important differences of PROs using in our manuscript is currently unknow. We mentioned this fact among the limitations of this paper (Line 778-786).

Statistics

1. Predictors cannot be identified in a cross-sectional study (lines 152-154)

Response: Thank you for the remind, we modified the presentation of this part (Line 195-196).

2. The model seems overloaded (table 3)

Response: We appreciate for your advice, and we simplified the model and described the results of univariate and multivariate linear regression analysis separately (table 3, table 4).

3. Multiple correlations are probably not the method of choice for analyzing between-group changes in the prospective study (lines 157-160).

Response: Thank you for the remind, we changed the statistical method from multiple correlations to linear regression analysis and we further assessed HRQoL using one-way analysis of variance (ANOVA) followed by pairwise comparisons according to the Least-Significant-Difference method (Line 213-218).

4. An expert statistician should be consulted with respect to analytical approach.

Response: Thank you for your advice, we consulted the expert statistician and corrected the deficiencies (Line 191-195, Line 213-218). In addition, we re-analyzed the data (Table 1, Table 2, Table 5, S1 Table).

Results

1. In general, I suggest dropping the use of two decimal places (text and tables). It indicates a precision level that is not real. Example: duration of symptoms 19.89 +27.34 months (line 168 + tab1).

Response: Thank you for your suggestion, we totally agreed that two decimal places indicates a precision level that is not real. We changed the use of two decimal places to one decimal place in all text and tables.

2. Tab 2. All variables have large SDs, simply reflecting the fact that wide ranges will always be found in very heterogeneous study populations. To what end is such information useful?

Response: We agreed that variables that have large SDs reflect the very heterogeneous populations in our study. So, we limited the study population to IIP and CTD-ILD patients and re-analyzed the data. The results for the two group was added.

3. In the longitudinal study, relatively large proportions of patients had improvements in both symptoms (dyspnea, cough) and PFTs, and also in HRQ scores. Patients with ILD tend to deteriorate or remain stable over time, not improve. Presumably the unexpected findings reflect that in a mixed study population - including patients with a broad variety of diagnoses/diseases - some will respond to therapy/drugs while others will not. (lines 246-257).

Response: Thank you for your comments. We agreed that the heterogeneous study populations in our study led to the unexpected findings, so we excluded Sarcoidosis, HP and other ILD subtypes in our analysis. After reanalyzing the data, we found that symptoms including dyspnea and cough deteriorated or remained stable or in most patients at follow-up (S1 table). At 6-month and 12-month follow-up, patients with IIP had a stable quality of life compared with baseline in HRQoL measured by SF-36 PCS, SGRQ total domains. However, the HRQOL in patients with CTD-ILD had a valuable improvement at 6-month and 12-month follow-up. The reason for the fact is likely to be the therapy or drugs. We are sorry for our negligence in this factor at follow-up and we explained this in the discussion (Line 792-793).

Reviewer #2:

The original research article by Yuan and co-authors assessed longitudinally the quality of life of 200 ILD patients at baseline, and at 6- and 12-month follow up correlating the changes in HRQoL with the changes in pulmonary function tests. The authors found that HRQoL deteriorates during the follow up of ILD patients and seems to be associated with determinants such as dyspnea, cough, and depression.

Overall the manuscript is well written and comprehensive; therefore I suggest only minor revisions.

1. Abstract – Introduction – Lines 16-18. The sentence “There are limited therapeutic options and strong side effects, which eventually lead to respiratory failure and affect the quality of life of patients” seems to suggest that the treatment-related side effects cause respiratory failure.

Response: Thank you for the remind, it is indeed our mistake in the expression. The sentence (Line 16-19) was modified.

2. Abstract – Results – Line 27. Correct “The mean age was 60.7 years old, …” with “The mean age was 60.7 years, …”.

Response: Thank you for pointing out the error, we have corrected it.

3. Introduction – Lines 65-68. I suggest quoting the few available studies dealing with HRQoL in ILD patients.

Response: Thank you for your advice, several available studies have been added in corresponding statements of the manuscript (**Line 81-84,** Respir Med. 2017; 127: 1–6, Respirology. 2018. https://doi.org/10.1111/ resp. 13293, Chest. 2014; 145: 1333–1338, J Bras Pneumol. 2010; 36: 562–570)

4. Results – Line 164. I suggest using the same number of decimals for the median age and its standard deviation, as it has been done in the whole manuscript for continuous variables.

Response: Thank you for your advice, we have unified all the median±SD to one number of decimals in the whole manuscript.

5. Results – Table 1. Correct “Ethmic group” with “Ethnic group”.

Response: Thank you for pointing out the error, we have corrected it.

6. Results – Lines 251-252. Does the sentence “Similarly, 42.4% of patients’ FVC% predicted and 48.8% of patients’ DLco% predict were improved or stable” refer to the 12-month follow up? If so, it should be specified in the text.

Response: Yes, thank you for your suggestion. We have made additional explanations in the text (Line 427-429).

7. Results – Lines 263-264. “The associations between longitudinal changes in HRQoL and clinical characteristics is shown in Table 4”. I suggest indicating in the text which clinical characteristics were tested in this analysis.

Response: We appreciate for your advice, and specific clinical characteristics including FVC% predicted, DLco% predicted, mMRC and LCQ total score have been described in the manuscript (Line 498-500).

8. Results – Line 284. Change “the emergence of the first symptom” with “the onset of the first symptom”.

Response: Thank you for your advice, we have corrected it.

9. Discussion. This paper is an example of the raising interest in the patient-reported outcome measures (PROs) in IPF and ILDs in general. However, the minimal clinically significant difference of the PROs used in this paper (mMRC, LCQ, HADS, SGRQ and SF-36 questionnaire) is currently unknown. This should be mentioned among the limitations of this paper.

Response: Thank you for your advice, we have elaborated this limitation in the discussion of the manuscript (Line 778-786).

We acknowledge the editors’ and reviewers’ comments and suggestions very much, which are

valuable in improving the quality of our manuscript. Thank you again for the guidance and

help.

Sincerely yours,

Xue-Yan Yuan, Ai Cui

2020-04-07
