# Supplementary material for: Evaluation of health-related quality of life and the related factors in a group of Chinese patients with interstitial lung diseases
Source: PLoS One. 2020 Jul 29;15(7):e0236346. doi: 10.1371/journal.pone.0236346 (PMC7417083; doi:10.1371/journal.pone.0236346)
Supplement: Attachment — Submitted filename: Response to Reviewers.docx [file pone.0236346.s005.docx]

Dear Prof. Paola Faverio,

Thank you for your kind letter. We have revised our manuscript after reading the comments and suggestions provided by you and the reviewers. The following is what we have modified in response to your and the reviewers’ comments and advice.

I agree with the minor revisions proposed by Reviewer 3.

I also suggest the authors to have the abstract of the manuscript, particularly the sentences changed during the review, rechecked by an English translator.

We appreciate all your and the reviewers’ comments and advice. According to your suggestion, we employed an English-language editing service, Academic English Editors (AJE) to polish our wording of the changed sentences and abstract, and the revised parts were marked in the red in the “Revised Manuscript with Track Changes”. We also elaborated on the diagnosis of the included patients in this study.

Responds to the reviewer’s comments:

Reviewer #3:

Xue-yan Yuan and collaborators wrote a research article trying to clarify the role of health-related quality of life factors in a cohort of Chinese patients affected by ILD.

This topic has been widely discussed in literature, however it is still a subject of interest.

Some minor revisions need to be made.

Methods

1. Line 80. In this section I suggest you to add which ILDs you include in your cohort (lately in the section Results you state you have considered only ILD patients with a diagnosis of CTD-ILD or IIP).

Response: Thank you for the comment and suggestion. The specific ILDs subtypes have been described in the manuscript (Line 84, Line 89, and Line 91: “Patients aged ≤85 years who were diagnosed with ILDs including IIP and CTD-ILD at the Department of Respiratory and Critical Medicine of Capital Medical University”, “The diagnosis of IIP or CTD-ILD”).

Results

1. Line 180 since idiopathic interstitial pneumonias include different entities as well as CTD-ILD (Am J Respir Crit Care Med. 2013 Sep 15;188(6):733-48) I suggest you to specify the composition of your cohort. This could help better understand your results since in both groups are included entities with different evolution and prognosis. Moreover it could help understanding why antifibrotic drug were administered in only 2.9% of the IPP group (were IPF patients the minority of this group?)

Response: Thank you for the remind. According to your suggestion, we have revised our manuscript and the specific composition of our included patients has been increased in the manuscript (Line 200-204: “139 were diagnosed with IIP (101 idiopathic non-specific interstitial pneumonia (NSIP) cases, 18 unclassifiable IIP cases, 11 respiratory-bronchiolitis-ILD cases, 7 IPF cases, and 2 others), and 30 were diagnosed with CTD-ILD (12 Sjögren’s syndrome (SS) cases, 6 undifferentiated CTD cases, 4 polymyositis/dermatomyositis (PM/DM) cases, 3 scleroderma (SSc) cases, 2 rheumatoid arthritis (RA) cases, and 3 others) (S1 Table)”).

2. Line 238. “ILD subtype (IIP vs CTD-ILD) was associated with most dimensions of the SGRQ and the SF-36 PCS” I suggest you to deepen this statement. Are there significant differences in the results of this questionnaires in IIP patients compared to CTD-ILD?

Response: Yes, patients with IIP had significantly better quality of life than patients with CTD-ILD in terms of SF-36 PCS, SGRQ activity, SGRQ impact, and SGRQ total domains in our study which were showed in Table 2. We appreciate your suggestion, and we have further elaborated the relevant information in the manuscript (Line 262-267: “As shown in Table 3, HRQoL was found to be significantly affected by multiple factors. ILD subtype was negatively associated with the SF-36 PCS and positively associated with most dimensions of the SGRQ in the univariate liner regression analyses. As previously mentioned, patients with CTD-ILD had a lower quality of life as measured by the SF-36 PCS and SGRQ activity, impact, and total domains when compared with patients with IIP”).

3. In section Results the comparison between IIP and CTD-ILD is clear only in the first and second paragraphs (patient characteristics and HRQoL) while in the other paragraphs (factors influencing HRQoL assessed at the time of enrollment; relationship between the change in HRQoL and changes in clinical characteristics and clinical characteristics and HRQoL of patients who died during follow-up) the comparison is absent. In these last paragraphs you consider “ILD subtypes” as a factor influencing or not HRQoL while I think it could be of interest analyze not only the whole cohort but also if there are differences between the two groups in terms of HRQoL.

Response: Thanks for your comments and suggestions. In this study, we mainly focused on the quality of life of all patients with ILDs and their influencing factors. Meanwhile, ILD subtypes were weakly associated with SF-36 PCS. So, we did not analyze the influencing factors in HRQoL of IIP and CTD-ILD patients, respectively. We compared the quality of life of IIP and CTD-ILD patients, and found HRQoL of patients with IIP was better than that of patients with CTD-ILD.

4. Moreover, a better focus on differences between the two groups should be highlighted in the section Discussion and in the section Conclusion.

Response: We appreciate for your advice and agree with the opinion. We had an in-depth discussion of the differences between the two groups. (Line 376-378, Line 402-409, Line 415, Line 419-424, Line 504-506)

Finally, we acknowledge the editor’ and reviewers’ comments and suggestions very much, which are valuable in improving the quality of our manuscript. Thank you again for the guidance and help.

Sincerely yours,

Xue-Yan Yuan, Ai Cui

2020-06-14
